# Disease Activity Assessment Frequency in Rheumatoid Arthritis: A Retrospective Observational Study of the Medical Support System for Rheumatoid Arthritis System Implementation

**DOI:** 10.2196/74222

**Published:** 2025-09-23

**Authors:** Mari Yamamoto, Yuki Kataoka, Yasushi Tsujimoto, Fumika Nagase, Keita Iwasaki, Yuuki Ito, Hiroki Ikai, Tsuyoshi Watanabe, Waka Yokoyama-Kokuryo, Naoho Takizawa, Yoshiro Fujita

**Affiliations:** 1 Department of Rheumatology and Nephrology, Chubu Rosai Hospital, 1-10-6 Komei, Minato-ku, Nagoya, Aichi, 455-8530, Japan, 81 526525511, 81 526533533; 2 Scientific Research WorkS Peer Support Group (SRWS-PSG), Osaka, Japan; 3 Infection Control Team, Chubu Rosai Hospital, 1-10-6 Komei, Minato-ku, Nagoya, Aichi, Japan, 81 52-652-5511; 4 Department of Internal Medicine, Kyoto Min-iren Asukai Hospital, Kyoto, Japan; 5 Department of International and Community Oral Health, Tohoku University Graduate School of Dentistry, Miyagi, Japan; 6 Department of Healthcare Epidemiology, Kyoto University Graduate School of Medicine/Public Health, Kyoto, Japan; 7 Oku Medical Clinic, Osaka, Japan; 8 Department of Health Promotion and Human Behavior, Kyoto University Graduate School of Medicine/School of Public Health, Kyoto University, Kyoto, Japan; 9 Division of Rheumatology, Department of Internal Medicine, Showa University School of Medicine, Shinagawa-ku, Tokyo, Japan

**Keywords:** disease management, electronic health records, quality improvement, rheumatoid arthritis, Medical Support System for Rheumatoid Arthritis, MiRAi

## Abstract

**Background:**

Rheumatoid arthritis (RA) is a global health concern with increasing prevalence. Despite recommendations for regular disease activity assessments, their implementation in routine clinical practice remains challenging. The Medical Support System for Rheumatoid Arthritis (MiRAi) is an offline, semi-automated system that calculates disease activity indices by integrating patient and clinician inputs from electronic health records (EHRs).

**Objective:**

This study evaluated the association between MiRAi implementation and the frequency of disease activity assessments in patients with RA.

**Methods:**

We conducted a retrospective cohort study of patients with RA treated at a tertiary hospital in Japan between April 2022 and March 2023. We included all adult outpatients (aged ≥18 years) with RA diagnosed according to the 2010 ACR/EULAR [American College of Rheumatology/European Alliance of Associations for Rheumatology] classification criteria. The hospital introduced MiRAi in June 2022 and achieved full deployment by October 2022. MiRAi calculated the clinical disease activity index (CDAI) and the modified health assessment questionnaire (mHAQ) through automated extraction of joint counts, patient global assessment, and functional status from structured EHR fields. Primary outcomes included the frequency of CDAI and mHAQ assessments. We administered a structured post-implementation survey to assess rheumatologists’ perceptions of MiRAi.

**Results:**

Physicians used MiRAi for 236/884 (26.7%) patients with RA. Patients with documented CDAI and mHAQ scores increased from 29 (5.9%) in June 2022 to 81 (19.0%) in November 2022, representing a 3.2-fold increase. Among surveyed rheumatologists (n=10), 5 (50%) reported the regular use of MiRAi. Physicians who regularly used MiRAi (n=5) cited improved accuracy in disease assessment and enhanced treatment decision-making. Non-users and occasional users (n=5) identified three primary barriers: limited familiarity with MiRAi, time constraints, and discrepancies between clinical judgment and MiRAi-generated outputs. Despite MiRAi’s availability, only 168 (19%) patients underwent quantitative disease activity assessment by the study end. Among 15 patients with high disease activity (CDAI >22), physicians recorded 3 treatment modifications and 2 intra-articular steroid injections.

**Conclusions:**

MiRAi implementation increased disease activity assessment frequency by 3.2-fold over 6 months; however, physician adoption remained at 26.7%, below the 80% target for routine care. Future implementation strategies should address identified barriers through system integration, structured user training, and workflow optimization to achieve guideline-concordant care for patients with RA.

## Introduction

Rheumatoid arthritis (RA) is a major global health concern. A 2021 meta-analysis, which included data from 67 facilities across 41 countries, reported a pooled RA prevalence of 0.46% (95% CI 0.37%‐0.57%) based on studies conducted between 1986 and 2014 [1]. A 2020 national survey in Japan estimated the prevalence of RA to be 0.75% [2]. The prevalence of RA varies across countries and is influenced by socioeconomic conditions, environmental exposures, and rheumatologist availability, as demonstrated in a global systematic review [3]. Despite regional differences, the global prevalence of RA continues to increase annually [4]. The assessment of RA activity requires both clinical and biological evaluations. Standard assessment tools for RA, such as the Clinical Disease Activity Index (CDAI) and Disease Activity Score in 28 Joints (DAS28), include subjective assessments [5]. Treatment adjustments based on disease activity are widely recommended [6].

DAS28 requires complex calculations, limiting its practicality in routine clinical settings, whereas CDAI is simpler to compute and demonstrates high utility [7]; however, published reports on their real-world application remain scarce and limited. A 2015 survey revealed that 59% of Japanese rheumatologists aimed to achieve clinical remission; however, only 45% used a composite measure of disease activity [8]. An online assessment tool has been developed for RA; however, it cannot be used if electronic health records (EHRs) are not connected to the Internet because of security concerns [9].

The treat to target (T2T) strategy—characterized by shared decision-making between patients and clinicians—effectively reduces disease activity and functional impairment [10]. However, a 2024 cross-sectional survey reported that most of the 166 rheumatologists perceived discordance between composite scores (eg, DAS28, CDAI) and clinical judgment, especially in cases with conflicting patient-reported outcomes [11]. This perceived misalignment and lack of consistent monitoring remain significant real-world barriers to T2T implementation. These concerns were echoed at the 2024 American College of Rheumatology and U.S. Food and Drug Administration joint summit, where experts highlighted critical limitations in RA disease activity assessments—including inconsistent definitions, non-standardized evaluation frequency, and difficulty in leveraging EHRs and insurance claims data for longitudinal monitoring [12]. The summit emphasized the need for the harmonization of assessment frameworks across real-world clinical environments.

The Medical Support System for Rheumatoid Arthritis (MiRAi) is an automated offline tool that integrates data from electronic health records to calculate objective assessment scores for collagen diseases. Since its development in 2009, the MiRAi has been implemented in 16 facilities across Japan. The use of MiRAi for RA disease activity assessment has been shown to improve patient satisfaction [13]; however, its impact on the frequency of assessment remains unclear.

We aimed to evaluate whether the implementation of MiRAi increased the frequency of assessment of disease activity in patients with RA.

## Methods

### Study Design and Participants

This retrospective cohort study was conducted at the Chubu Rosai Hospital, a tertiary-care facility in Japan, and included patients with RA who visited the Rheumatology Department between April 2022 and March 2023. We identified patients from the outpatient database and included them consecutively based on diagnostic codes to reflect routine clinical practice. The primary physician confirmed each RA diagnosis and enrolled the patients. No additional interventions were introduced during the observation period. In this institute, the operational use of MiRAi began in June 2022. After integrating MiRAi into the EHR system, we began using it in an outpatient setting. Initially, a few physicians used the MiRAi on a trial basis. In October 2022, all 10 staff members were informed about the system, and MiRAi was fully implemented.

### The MiRAi System

MiRAi, developed by a Japanese medical institution in 2009, is an electronic system that automatically provides RA activity indicators after patient and physician assessments are entered into an attached tablet and EHR. The MiRAi system operates as follows. First, the patient enters the patient visual analog scale (VAS) and modified health assessment questionnaire (mHAQ) on a tablet in an outpatient setting. Next, during the consultation, the physician clicks on the MiRAi tab, and the values entered by the patient are reflected in the EHR. The physician then enters the doctor’s VAS, C-reactive protein level, and erythrocyte sedimentation rate, following which the DAS28 and CDAI are automatically calculated. The locations of the swollen and tender joints are entered by clicking on the diagram, and the previously recorded data can also be reviewed.

### Outcomes

To verify the changes in disease activity frequency following the introduction of MiRAi, we assessed the following factors: the proportion of CDAI and mHAQ evaluations among unique patients with RA per month; for patients with active RA (CDAI >22), the percentage of patients who changed or added targeted synthetic disease-modifying antirheumatic drugs, biological DMARDs, or conventional synthetic DMARDs; and the proportion of patients who received steroid joint injections during the follow-up period.

### Questionnaire Survey and Data Analysis

In April 2024, we conducted a questionnaire survey based on the behavior change stage model [14]. The survey was administered to physicians who provided care at the outpatient clinic of the Rheumatology Department. The survey included multiple-choice questions regarding the disease activity indicators used for RA and the use of MiRAi. Information regarding the advantages and disadvantages of MiRAi was collected from physicians who reported using it, while non-users were requested to state their reasons for not adopting MiRAi. We performed descriptive statistics to analyze the data using R Studio (Posit PBC, Boston, MA, USA) [15].

### Ethical Considerations

The study adhered to the World Medical Association Declaration of Helsinki ethical guidelines [16] and the Ethical Guidelines for Medical and Biological Research Involving Human Subjects [17]. The Chubu Rosai Hospital Ethics Committee approved the study protocol (approval number: 202102‐06). We obtained written informed consent from all participants before enrollment. To ensure confidentiality, we de-identified all data before analysis. Participants did not receive any compensation or incentives. As the manuscript does not include any images or supplementary materials containing identifiable information, we did not require additional consent for image publication.

## Results

### Patient Characteristics

During the study period, 884 patients with RA visited our outpatient clinic, with an average of approximately 480 visits per month. MiRAi was used in 236 patients, accounting for 26.7% of all patients with RA. This retrospective observational study evaluated changes in disease activity assessment frequency following MiRAi implementation in routine clinical practice. MiRAi was introduced in June 2022, and full implementation began in October 2022.

### Evaluation of Disease Activity and Therapeutic Interventions for Patients

**Figure 1.**
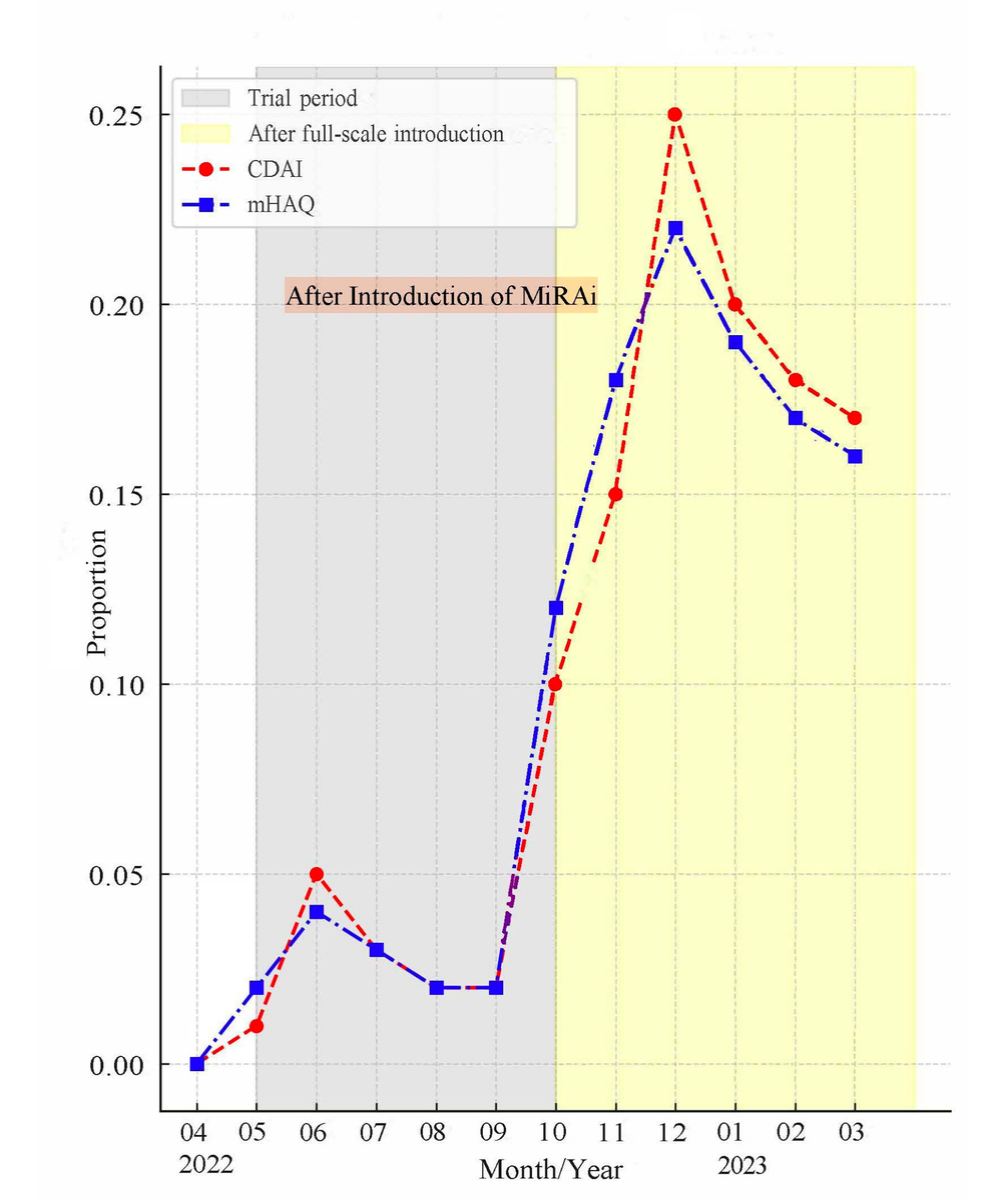
Monthly proportion of patients with RA who underwent CDAI and mHAQ assessments before and after implementation of the MiRAi system at Chubu Rosai hospital (April 2022–March 2023). RA: rheumatoid arthritis; CDAI: clinical disease activity index; mHAQ: modified health assessment questionnaire; MiRAI: Medical Support System for Rheumatoid Arthritis.

**Figure 2.**
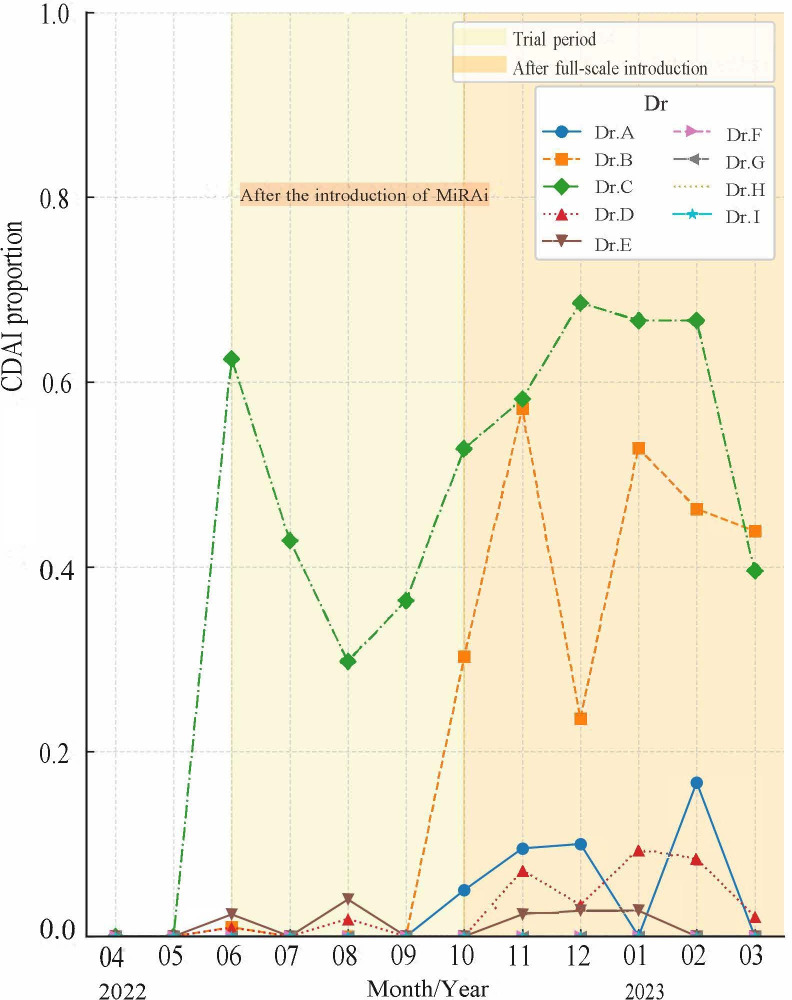
Proportion of patients with RA who underwent CDAI assessment by individual physicians at Chubu Rosai hospital during the post-implementation period of MiRAi (June to November 2022). This figure illustrates the variability in MiRAi usage across physicians. Differences may reflect variations in familiarity with the system, time constraints during outpatient visits, and attitudes identified in the post-implementation physician survey. The study was conducted as a retrospective observational analysis in a single tertiary-care facility in Japan. RA: rheumatoid arthritis; CDAI: clinical disease activity index; MiRAI: Medical Support System for Rheumatoid Arthritis.

**Figure 3.**
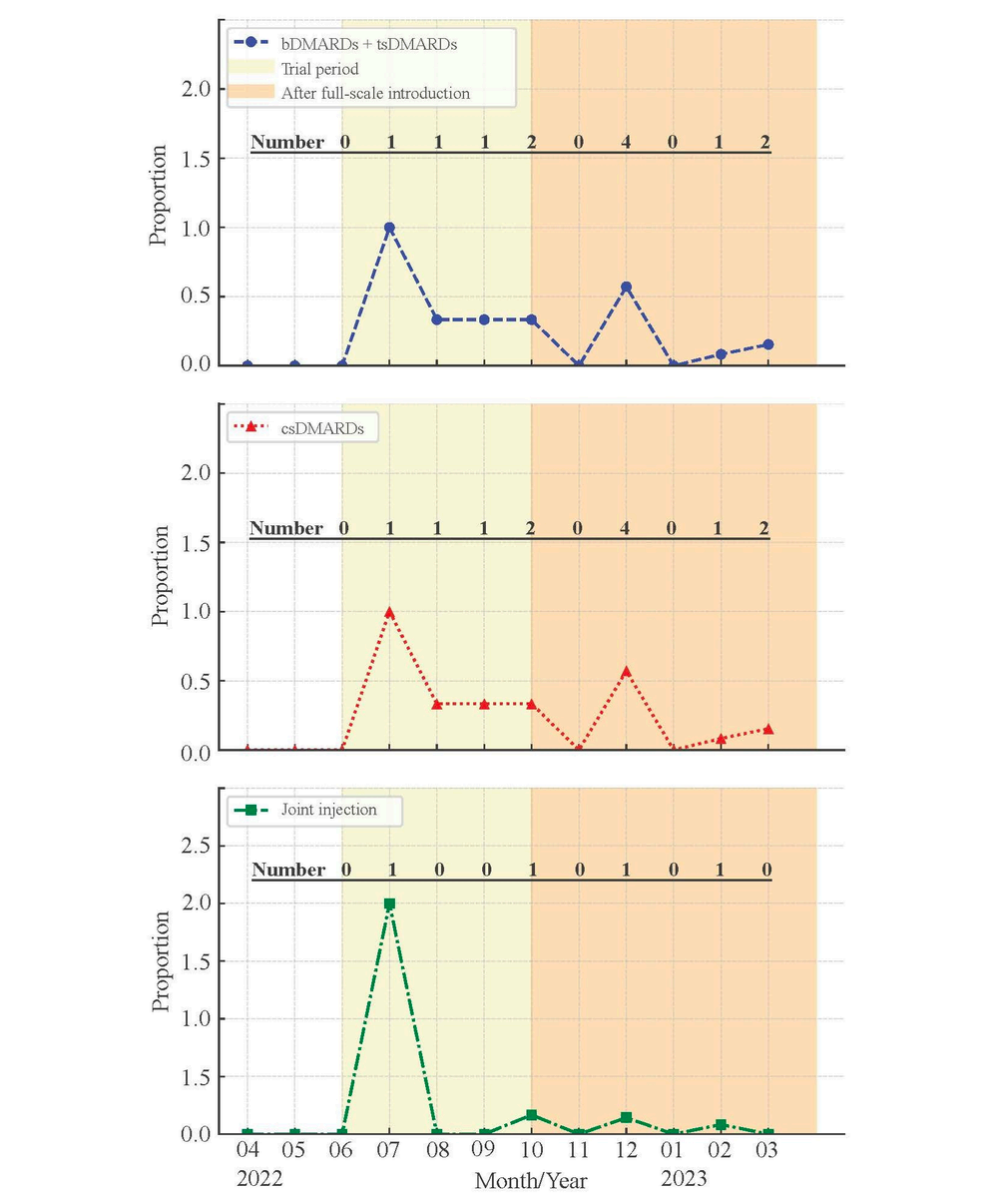
Proportion of patients with active rheumatoid arthritis (RA) (CDAI >22) who underwent treatment modifications—such as changes or additions of targeted synthetic disease-modifying antirheumatic drugs (tsDMARDs), biological DMARDs (bDMARDs), or conventional synthetic DMARDs (csDMARDs)—and those who received intra-articular steroid injections during the follow-up period (April 2022–March 2023). This figure reflects treatment response trends among patients with high disease activity in the context of MiRAi implementation at a tertiary-care hospital in Japan. Due to the limited number of high-activity cases, the number of events was small.

Before the introduction of MiRAi, no quantitative evaluations of disease activity were performed at our facility. The number of CDAI and mHAQ evaluations increased from 29 (5.9%) in June 2022 during the trial period to 81 (19%) in November 2022 after the full implementation of MiRAi (Figure 1). Additionally, the percentage distribution of MiRAi ratings assigned to each physician was analyzed (Figure 2). We performed treatment evaluations in patients with high RA activity and those with RA remission. The number of events did not exceed 5 (Figure 3).

### Questionnaire Survey

We mailed the survey to 10 rheumatologists affiliated with the Collagen Disease Department at Chubu Rosai Hospital, and all 10 responded. Among them, 70% (7/10) had over 4 years of experience in treating collagen diseases, while 30% (3/10) had less than 3 years of experience. Half of the rheumatologists believed that their disease activity assessments were appropriate (5/10), 40% (4/10) were not confident, and 10% (1/10) were unsure. Additionally, 50% (5/10) of the physicians reported regular use of MiRAi. The results of the content analysis are presented in Table 1.

**Table 1.** Post-implementation survey results.

Category	Negative comments	Positive comments
System	I do not understand how to use the system. Using MiRAi^a^ takes time. I want to be able to easily attach joint diagrams to medical records. I want to be able to easily make reservations for equipment. I want detailed explanations on how to use it.	It was convenient after using it.
Evaluation method	I think other evaluation methods are sufficient. There is a discrepancy between MiRAi’s evaluation and my own activity assessment. It is hard to grasp the degree of joint swelling.	I thought about improving the RA^b^ evaluation method. It allows for more accurate assessments of disease activity. Deciding on treatment plans became easier. Communication with patients became smoother.
Information sharing	I want a mechanism to explain, distribute, and introduce MiRAi for all patients with RA. I do not have time to explain the introduction of MiRAi to patients.	It became easier to share information with other medical staff.

a MiRAi: Medical Support System for Rheumatoid Arthritis.

b RA: rheumatoid arthritis.

## Discussion

### Principal Findings

In this retrospective cohort of 884 patients with RA at Chubu Rosai Hospital (April 2022–March 2023), MiRAi use rose to 26.7%, and the combined CDAI/mHAQ assessment proportion increased from 5.9% (June 2022) to 19% (November 2022). Among those with high activity (CDAI >22), targeted DMARD adjustments and steroid injections were recorded, though infrequently. In a survey of all 10 rheumatologists, 50% regularly used MiRAi—citing its convenience, assessment accuracy, and smoother patient communication—while noting time demands, usability hurdles, and training needs. These results suggest that MiRAi integration boosts RA monitoring but requires refinements to optimize clinician uptake. These results warrant further interpretation in the context of existing RA literature.

Despite system-wide availability, MiRAi was used in only 19% of patients during the study period. A post-observation survey of 10 rheumatologists revealed that three main barriers limited adoption: unfamiliarity with the system, time constraints, and perceived discrepancies between clinical judgment and MiRAi outputs. Conversely, half of the physicians reported the regular use of MiRAi and identified three major benefits: improved clarity in disease activity assessment, enhanced support for treatment decisions, and more effective patient communication.

### Interpretation and Comparison With Prior Works

To our knowledge, this is the first study to examine the integration of MiRAi, a semi-automated system, into routine clinical practice. We investigated whether its use is associated with changes in the frequency of RA disease activity assessment. In a clinical setting with outpatient visits every 1‐3 months, approximately 20% of patients underwent quantitative assessments within 1 year of MiRAi implementation. This finding contrasts with those of previous studies that reported less frequent assessments. A multinational registry of routine RA care (METEOR) includes prospectively collected data from both academic and community rheumatology practices across 23 countries. It revealed that 85.9% of patients had at least one documented disease activity assessment over 4 years [9]. Additionally, a nationwide analysis of U.S. insurance claims data from commercially insured and Medicare Advantage patients with newly diagnosed RA highlighted substantial gaps in care quality. Only 44.1% of patients visited a rheumatologist within 1 year of diagnosis, and 31.3% met none of the recommended quality care indicators—such as annual clinical assessments and routine laboratory testing [18]. These results suggest that, particularly in routine care settings, routine disease activity assessments remain suboptimal. The semi-automated nature of MiRAi might improve the frequency of disease activity assessments compared with previous findings.

### Clinical and Research Implications

The use of MiRAi might be associated with increased frequency of disease activity assessments, which in turn could support more standardized and objective monitoring in routine RA care. However, its clinical impact on treatment decisions and patient outcomes remains uncertain. In our study, MiRAi was used in only 81 patients (19%) during the observation period, and the number of patients with high disease activity or remission was small. As a result, we were not able to evaluate whether increased assessment frequency led to treatment modifications or changes in clinical outcomes.

Given these observations, further research is warranted. Future multicenter studies with larger sample sizes and longer follow-up are needed to clarify whether continued use of MiRAi contributes to timely treatment adjustments, better disease control, and improved long-term outcomes in patients with RA. In addition, barriers to adoption—such as usability issues, time constraints, and perceived discrepancies between clinician judgment and system outputs—must be addressed. Similar challenges have been reported in other clinical decision support system implementations, where limited integration into clinical workflows, lack of transparency, and insufficient personalization have been identified as key factors impeding adoption [19]. These insights underscore the broader need for thoughtful integration of digital tools into clinical workflows, ensuring usability, trust, and alignment with clinical needs to maximize their potential impact.

### Limitations

Our study had some limitations. After the introduction of the system, only half of the physicians conducting outpatient consultations adopted MiRAi. Furthermore, the extent of improvement in disease assessment frequency among physicians using the system varied significantly. To investigate the reasons for this limited adoption, we conducted a post-observation survey of physicians and performed content analysis, which revealed several key findings. First, some physicians initially did not know how to use MiRAi. This indicates that the explanation provided during the introduction of the system was insufficient, underscoring the need for accessible user manuals. Second, many physicians rely on swollen joint counts, tender joint counts, and C-reactive protein levels for evaluation [20]. In contrast, half the physicians admitted that they lacked confidence in their disease activity assessments during consultations. This may be due to the absence of clear indicators of disease activity in routine clinical practice. Some physicians noted the benefits of MiRAi, including more accurate disease activity assessments and improved decision-making regarding treatments. These findings suggest that MiRAi can serve as a valuable tool for conducting appropriate disease activity evaluations. Many physicians who did not regularly use MiRAi cited time constraints, indicating that integrating the new system within a limited consultation time may have been burdensome for outpatient doctors. Therefore, it is necessary to automate the system to seamlessly incorporate objective evaluations into daily clinical practice. Additionally, tablet-based input by all patients with RA, without requiring explanations from attending physicians, could help reduce the clinicians’ workload.

In addition, the single-center design and limited MiRAi adoption (19% of patients with RA) constrain generalizability. Institutional factors such as staffing, EHR integration, and patient characteristics may affect implementation in other settings. These findings should be interpreted cautiously, and future multicenter studies are planned to evaluate MiRAi’s utility, adoption, and clinical impact across diverse care environments. Lastly, although MiRAi is designed to support both clinicians and patients, this study did not include an evaluation of patient perspectives. Understanding patient satisfaction, perceived usefulness, and the system’s impact on shared decision-making is essential to fully assess its clinical value. We acknowledge this as a limitation and are currently planning a follow-up study that incorporates a patient questionnaire to address this important aspect.

### Conclusions and Broader Implications

The implementation of MiRAi might increase the frequency of RA disease activity assessment. Future multicenter studies with sufficient sample sizes using MiRAi are warranted to verify whether the continued quantitative assessment of patients with RA leads to appropriate treatment changes.

## Abbreviations

ACR/EULAR: American College of Rheumatology/European Alliance of Associations for Rheumatology
bDMARDs: biological disease-modifying antirheumatic drugs
CDAI: Clinical Disease Activity Index cs
DAS28: Disease Activity Score in 28 Joints
DMARDs: conventional synthetic disease-modifying antirheumatic drugs
DMARDs: targeted synthetic disease-modifying antirheumatic drugs
EHR: electronic health record
mHAQ: modified Health Assessment Questionnaire
MiRAi: Medical Support System for Rheumatoid Arthritis
RA: rheumatoid arthritis ts
T2T: treat to target
VAS: visual analog scale
